# The Rebirth of Matrix Metalloproteinase Inhibitors: Moving Beyond the Dogma

**DOI:** 10.3390/cells8090984

**Published:** 2019-08-27

**Authors:** Gregg B. Fields

**Affiliations:** 1 Institute for Human Health & Disease Intervention, Department of Chemistry & Biochemistry, and the Center for Molecular Biology & Biotechnology, Florida Atlantic University, Jupiter, FL 33458, USA; fieldsg@fau.edu; Tel.: +1-561-799-8577; 2 Department of Chemistry, The Scripps Research Institute/Scripps Florida, Jupiter, FL 33458, USA

**Keywords:** matrix metalloproteinase, protease inhibitor, cancer, arthritis, wound healing

## Abstract

The pursuit of matrix metalloproteinase (MMP) inhibitors began in earnest over three decades ago. Initial clinical trials were disappointing, resulting in a negative view of MMPs as therapeutic targets. As a better understanding of MMP biology and inhibitor pharmacokinetic properties emerged, it became clear that initial MMP inhibitor clinical trials were held prematurely. Further complicating matters were problematic conclusions drawn from animal model studies. The most recent generation of MMP inhibitors have desirable selectivities and improved pharmacokinetics, resulting in improved toxicity profiles. Application of selective MMP inhibitors led to the conclusion that MMP-2, MMP-9, MMP-13, and MT1-MMP are not involved in musculoskeletal syndrome, a common side effect observed with broad spectrum MMP inhibitors. Specific activities within a single MMP can now be inhibited. Better definition of the roles of MMPs in immunological responses and inflammation will help inform clinic trials, and multiple studies indicate that modulating MMP activity can improve immunotherapy. There is a U.S. Food and Drug Administration (FDA)-approved MMP inhibitor for periodontal disease, and several MMP inhibitors are in clinic trials, targeting a variety of maladies including gastric cancer, diabetic foot ulcers, and multiple sclerosis. It is clearly time to move on from the dogma of viewing MMP inhibition as intractable.

## 1. Introduction

**Figure 1:**
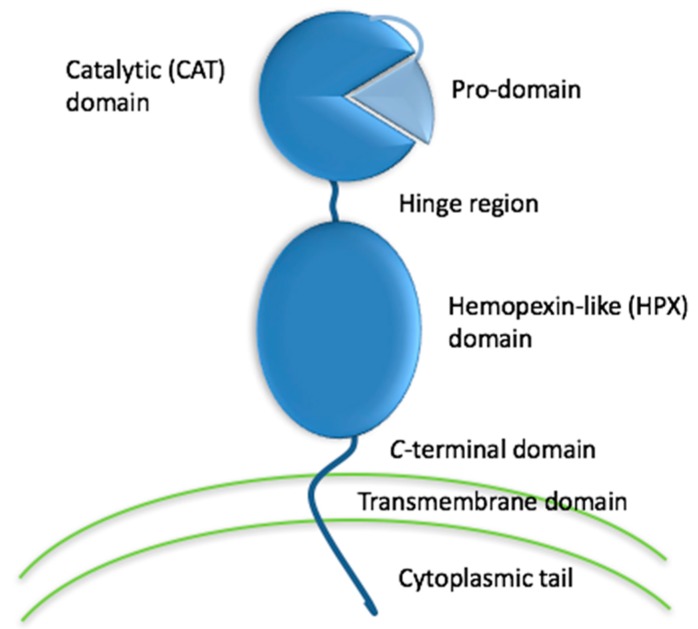
Schematic depiction of the domains of a representative MMP family member, membrane type 1 matrix metalloproteinase (MT1-MMP).

Activities of the matrix metalloproteinase (MMP) family (Figure 1) have long been correlated to disease initiation and progression [1,2,3,4,5,6]. For example, collagenolytic activity had been observed in tumor tissues, cells, and extracts since the late 1960s [7,8,9,10,11,12]. Initially, MMPs were considered as potential targets in cancer and osteoarthritis [13,14,15,16]. Subsequently, roles for MMPs emerged in neurodegenerative, infectious, and cardiovascular diseases [17]. MMPs can be present in three forms, latent (zymogen), activated, and activated but in complex with natural inhibitors such as tissue inhibitor of metalloproteinases (TIMPs). During conditions where active MMPs are overproduced, the regulation by TIMPs may be overwhelmed [18]. Developing inhibitors of the transcription, activation, or activity of MMPs thus represents an attractive therapeutic prospect [19].

Members of the MMP family are also essential for the maintenance of healthy processes, such as wound healing (e.g., MMP-1, MMP-2, MMP-3, MMP-8, MMP-9, MMP-10, MMP-13, and MT1-MMP), inflammatory response (e.g., MMP-2, MMP-3, MMP-7, MMP-8, MMP-9, MMP-10, MMP-12, MT1-MMP, MMP-19, and MMP-28), and angiogenesis (e.g., MMP-2, MMP-9, and MT1-MMP) [5,20,21,22,23]. Thus, inhibiting all MMP family members at the same time would be detrimental, as indicated by early clinical trials conducted with broad spectrum MMP inhibitors [24,25]. In addition to problems of selectivity, the low bioavailability and poor metabolic profile of broad spectrum MMP inhibitors led to limited beneficial effect and thus did not justify the further pursuit of clinical trials [18]. Overall, the previous poor performance of MMP inhibitors in clinical trials has been attributed to (a) inhibition of other metalloenzymes, (b) lack of specificity within the MMP family, (c) poor pharmacokinetics, (d) dose-limiting side effects/toxicity, (e) in vivo instability, and (f) low oral availability/inability to assess inhibition efficacy [26,27]. New classes of MMP inhibitors need to address these factors. In addition, as the role of MMPs may change from detrimental to beneficial, or vice versa, during the course of disease, the mechanism and/or substrate profile of MMPs needs to be clearly identified when designing clinical trial protocols. Finally, there needs to be a precise understanding as to which MMPs are relevant in specific diseases, and the forms (active versus inactive) that these MMPs may be present in.

## 2. Problems Associated with MMP Inhibition, Real and Imagined

### 2.1. MSS Induced by Broad Spectrum Inhibitors

Initial strategies for designing MMP inhibitors focused on the necessity of Zn^2+^ in the catalytic mechanism [14,28]. Small molecules such as hydroxylamine, which has the property of chelating Zn^2+^, were utilized as templates to create inhibitors with strong Zn^2+^ chelating properties [13,14]. Unfortunately, clinical trials using broad spectrum inhibitors often led to severe side effects and had to be discontinued [18,29,30]. More specifically, the poor selectivity of broad spectrum inhibitors resulted in musculoskeletal triad (arthralgia, myalgia, tendinitis)/musculoskeletal syndrome (MSS) and gastrointestinal disorders [31]. Development of roughly thirty MMP inhibitor-based anti-arthritic drugs were discontinued in clinical trials due to the occurrence of MSS [32,33,34].

MSS does not appear to be caused by the off-target inhibition of a single enzyme, but rather inhibition of a combination of several MMPs and/or possibly other related enzymes [35]. Among enzymes initially proposed to play role in MSS development were MMP-1 [35], MMP-2 [36], MMP-9 [37], MT1-MMP [38], and ADAM family members [35]. MSS was also attributed to combined inhibition of MMP-1 and ADAM17 [39] or ADAM10 and ADAM17 [30]. A pyrimidine-2,4,6-trione derivative that inhibited MT1-MMP, MMP-2, and MMP-9 was not associated with MSS [40], and no MSS was observed in animals treated with the MMP-13 selective inhibitor ALS 1-0635, even at an ALS 1-0635 concentration 200-fold greater than that of marimastat known to induce this condition [41]. Thus, MMP-2, MMP-9, MMP-13, and MT1-MMP are no longer considered to be involved in MSS. MSS has been evaluated in rat models, which may have certain caveats (as rodents have MMP-1a as a homolog to human MMP-1 [42]), but broad spectrum MMP inhibition results in fibroproliferation affecting the patellar tendon and other intra- and periarticular connective tissues in rats, mimicking MSS.

### 2.2. Off-Target Interactions of Hydroxamic Acid-Based Inhibitors

Hydroxamic acid-based inhibitors designed for MMPs, such as BB-94, were found to inhibit ADAM family members, such as ADAM17, more potently then MMPs [43]. Hydroxamic acids have poor selectivity for Zn^2+^ over other divalent first row transition metals [44]. Activity-based protein profiling probes [45] were designed based on GM6001, a hydroxamic acid that chelates Zn^2+^ and was thought to selectively inhibit MMPs. When analyzing the metalloproteinases increased in invasive melanoma, the enzymes that were affinity-purified were neprilysin (an integral membrane metalloproteinase), leucine aminopeptidase, and dipeptidylpeptidase III [45]. These results suggest that nonspecific inhibition of metalloproteinases (not limited to the MMP family) may explain the toxicity observed with MMP inhibitors in clinical trials [33,46,47].

A strong chelator does not necessarily result in nonspecific metalloenzyme inhibition [48]. A variety of chelators with greater selectivity for Zn^2+^ (compared with hydroxamic acids) have been developed as MMP inhibitors [48,49].

### 2.3. Metabolic Instability of Hydroxamic Acid-Based Inhibitors

In addition to a lack of selectivity, hydroxamic acids are problematic compounds due to their susceptibility to a variety of metabolic events. Hydroxamic acids can be hydrolyzed to carboxylic acids [50,51], which are typically less active than the parent compound. In addition, hydrolysis results in the production of hydroxylamine, a toxic byproduct [52]. Hydroxamic acids may also be reduced to amides [53], or *O*-glucuronylated or *O*-sulfated [51,54,55]. Hydroxamic acid stability in plasma is dependent upon esterase activity [56], specifically arylesterases and carboxylesterases [57]. The stability of hydroxamic acid-containing compounds is dependent upon (a) the accessibility of the esterase to the hydroxamic acid, (b) favorable or unfavorable interactions of the esterase with other constituents present in the compound, (c) compound hydrophilicity (logP), and (d) number of hydrogen bond donors in the compound [57]. Significant differences have also been noted in hydroxamic acid stability in rat versus human plasma [57].

### 2.4. Animal Models

Genetically modified (knockout) mice have been utilized to delineate the roles MMPs in normal physiological function and pathological states. Unfortunately, the identification of passenger mutations that accompanied MMP knockouts raised serious concerns as to the interpretation of results from disease models in which MMPs were implicated [58]. For example, the protection of *Mmp7*, *Mmp8*, or *Mmp13* null mice from lipopolysaccharide lethality [59,60,61] may well be due to a *Casp11*-inactivating passenger mutation [58,62].

The passenger mutation effect may lead to questions about knockout animal results when, in fact, the results have been interpreted correctly. For example, MMP-8 knockout mice were used to determine the role of MMP-8 in wound healing [63]. MMP-8 was found to contribute to the resolution of inflammation and promote re-epithelization, aiding wound repair [63]. Transplantation of bone marrow from wild-type animals restored normal wound healing in MMP-8 knockout mice [63]. As indicated above, interpretation of activity alterations in *Mmp8* null mice may be complicated due to *Casp11* inactivation. However, the positive contribution of MMP-8 to diabetic wound healing was confirmed by chemical inhibition approaches [20,64].

High levels of MMP-8 have been correlated with fatal outcomes in sepsis [65,66]. Subsequently, MMP-8 knockout mice demonstrated increased survival compared to wild-type animals when subjected to the cecal ligation and puncture (CLP) model of sepsis [66]. Survival was also improved upon the application of an MMP inhibitor ((3R)-(+)-[2-(4-methoxybenzenesulfonyl)-1,2,3,4-tetrahydroisoquinoline-3-hydroxamate] or GlyΨ{PO_2_H-CH_2_}Ile-His-Lys-Gln THPI) to wild-type mice subjected to CLP [66,67], and transplantation of bone marrow from wild-type animals into MMP-8 knockout mice subjected to CLP compared with transplantation of bone marrow from MMP-8 knockout mice into wild-type animals subjected to CLP [68]. In the case of inhibition, the inhibitory compounds may not have been selective for just MMP-8. The effect in sepsis may be due to a combination of, for example, MMP-8 and MMP-13 inhibition. Along these lines, a bispecific nanobody that inhibited MMP-8 and tumor necrosis factor receptor 1 offered complete protection in mice subjected to endotoxemia and CLP [69].

Comparison of MMP-9 knockout and wild-type mice found 34 plasma glycoproteins significantly different between the two, including Ecm1, periostin, and fibronectin [70]. The differing proteome background between the MMP-9 knockout and wild-type mice suggested that disease models utilizing pharmacological inhibition versus knockout of target enzymes may have different downstream results [70]. Indeed, it was found that CD36 (a phagocytic marker in macrophages) was reduced post-myocardial infarction in animals treated with an MMP-9 inhibitor but increased post-myocardial infarction in MMP-9 knockout mice [70]. It is important to note that in the knockout mice, MMP-9 was completely removed, while the MMP inhibitor reduced MMP activity by 30% [70]. Such differences in relative MMP-9 inhibition, as well as the aforementioned differences in proteome background, can produce different outcomes in the two animal model systems.

An additional concern with the application of MMP knockout mice for disease models are compensatory effects on other MMPs. For example, in the aforementioned study of the role of MMP-8 in wound healing, MMP-8 knockout mice were found to have increased levels of MMP-9 in the wound area compared with wild-type mice [63]. In contrast, the expression pattern of MMP-13 had a more restricted distribution in the MMP-8 knockout mice compared with the wild-type mice [63]. MMP-13 knockout mice exhibited enhanced expression of MMP-8 in wound areas compared with wild-type mice [71]. These compensatory effects complicate interpretation of results in which inhibitor-treated wild-type animals are compared with knockout animals.

Beyond knockout studies, there are examples of where initial interpretation of animal models has provided incorrect results for evaluating the roles of MMPs in disease. Based on animal studies, MMP-9 was viewed as an appropriate target for modulating colitis [72]. Clinical trials of a monoclonal antibody that inhibits MMP-9 (GS-5745/andecaliximab) for ulcerative colitis (UC) were subsequently undertaken [73,74]. A later study utilizing three different mouse models of colitis indicated that MMP-9 upregulation was a consequence, rather than a cause, of intestinal inflammation [75]. Clinical remission or response was not observed in the UC trial [74]. In addition to the initial colitis animal model results being misleading, the lack of success of GS-5745/andecaliximab in the UC clinical trial may also have been due to the antibody having higher affinity for proMMP-9 (K_D_ = 0.008–0.043 nM) compared with active MMP-9 (K_D_ = 2.0–6.6 nM) [76], and thus much of the applied inhibitor may not have been bound to the active form of the enzyme.

### 2.5. The Complexity of the Protease Web

**Figure 2:**
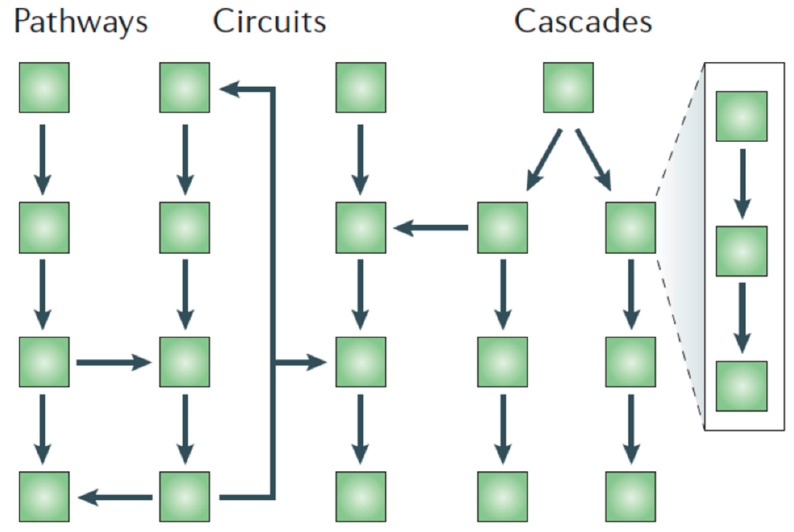
The “protease web”, in which proteases act in linear pathways, amplification cascades (with a one-way flow of information), or in circuits (with information feedback) [33]. Figure reproduced with permission of the Nature Publishing Group.

MMPs operate in linear pathways (direct substrates), amplification cascades, and regulatory circuits, resulting in a complex “protease web” (Figure 2) [33]. Therapeutic intervention should restore a normal MMP protease web, but is daunting in terms of possible side effects due to MMP inhibition [77]. It has been recommended that a systems biology approach be utilized to predict consequences of MMP inhibition [77]. Although many substrates have been identified and validated for MMPs [17,78], the kinetics for substrate turnover are often not reported. In addition, in vitro activity towards a substrate does not automatically indicate the processing of the substrate occurs in vivo. As has been noted, although MMPs can process chemokines, lack of proteolysis of a specific chemokine has not been observed in MMP knockout mice [79]. Since chemokine substrates of MMPs are often cleaved by multiple proteases, proteolytic redundancy may explain why chemokines are still processed in MMP knockout mice.

MT1-MMP has a significant role in angiogenesis, whereby it can exhibit both pro-angiogenic and anti-angiogenic behaviors [80,81,82,83,84,85,86,87,88]. These contrasting behaviors point to the importance of spatial and temporal expression of MT1-MMP. Similar concerns have arisen for MMP inhibition following ischemic stroke, where MMPs can disrupt the neurovascular system and blood–brain barrier, leading to cell death and cerebral edema, but can also contribute to neurorepair via angio-vasculogenesis, gliogenesis, and neurogenesis [89]. The same is true in neurological infectious diseases and multiple sclerosis, where inhibition during acute inflammation can be beneficial while inhibition during repair processes can be detrimental [6].

The complexity of the protease web is not necessarily a deterrent for inhibitor development. Angiotensin-converting enzyme (ACE) is well recognized for converting angiotensin I to angiotensin II and degrading bradykinin. ACE has an extensive substrate profile [90], including degrading acetyl-Pro-Gly-Pro. Acetyl-Pro-Gly-Pro serves to reduce neutrophilic inflammation and matrix degradation in chronic obstructive pulmonary disease (COPD) [91]. ACE also converts β-amyloid protein 42 (Aβ42, which is neurotoxic) to Aβ40 (which is neuroprotective) [92]. Despite a complex protease web for ACE, clinical applications of ACE inhibitors have existed for decades. A recent concern has arisen based on ACE inhibition resulting in increased brain amyloid deposition and apoptotic neurons in mice [93]. Whether this translates to human disease remains to be seen.

## 3. Development of Improved MMP Inhibitors

### 3.1. Creating Selective MMP Inhibitors

**Figure 3:**
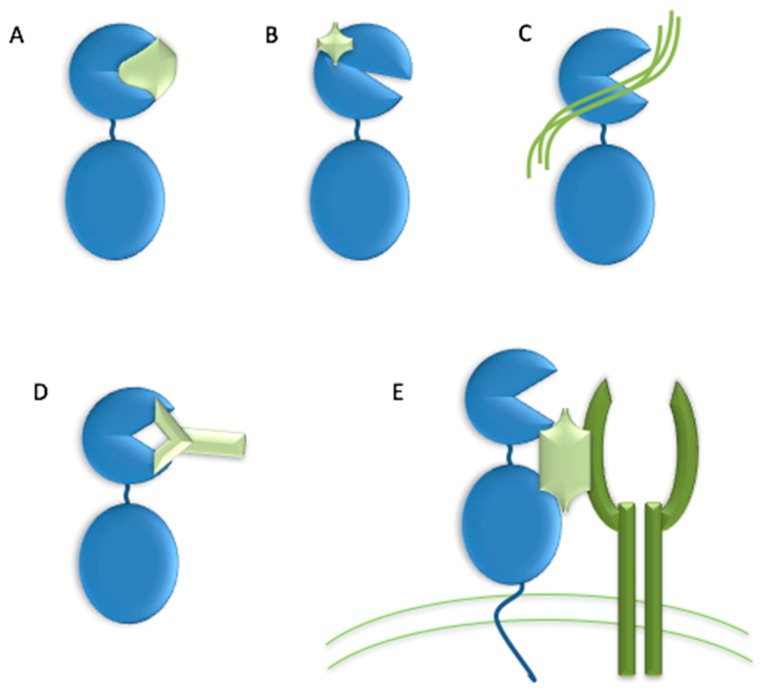
Strategies for creating selective MMP inhibitors. (**A**) endogenous-like inhibitors, (**B**) exosite targeting inhibitors, (**C**) combination of exosite binding and metal chelating inhibitor, (**D**) function blocking antibodies, and (**E**) inhibitor disrupting MMP interactions with cell surface binding partners.

The high homology within the MMP family has made the development of selective inhibitors difficult. Ideally, an MMP inhibitor should exhibit a ~3 orders of magnitude difference in K_i_ between the target MMP and other MMPs [32,48]. Numerous strategies have been suggested for creating selective MMP inhibitors, including (a) application of endogenous-like inhibitors (TIMP analogs), (b) exosite targeting (i.e., HPX domain or MMP-2/MMP-9 fibronectin type II inserts), (c) combination of exosite binding and metal chelation, (d) function blocking antibodies, and (e) disrupting MMP interactions with cell surface binding partners (Figure 3) [94].

Modification of TIMPs has been explored in an attempt to design selective MMP inhibitors [95]. It was initially observed that mutation of Thr2 to Ala in TIMP-1 resulted in an inhibitor that was much less effective towards MMP-1 compared with MMP-2 and MMP-3 [96] Additional mutations of Thr2 furthered the concept that TIMPs could be selectively targeted [97]. Mutation of residues 2, 4, and/or 68 produced a TIMP-1 variant (Thr2Arg/Val4Ile) that effectively inhibited MMP-2 and MMP-3, but did not inhibit MMP-1 [98]. In turn, mutation of Thr98 to Leu converted TIMP-1 to an effective inhibitor of MT1-MMP [99], although multiple substitutions were required to fully explain why wild-type TIMP-1 was a poor inhibitor of MT1-MMP [99,100]. The TIMP-1 triple mutant Val4Ala/Pro6Val/Thr98Leu, a very effective inhibitor of MT1-MMP (K_i_ = 1.66 nM) [99], was fused to a glycosyl-phosphatidyl inositol anchor for targeting to the cell membrane [101]. Mutation of Thr2 to Gly resulted in a TIMP-1 variant with virtually complete selectivity between MMP-9 (K_i_ = 2.1 nM) and MMP-2 (K_i_ > 40 μM), while mutation of Thr2 to Arg and replacement of the TIMP-1 AB loop with the TIMP-2 AB loop produced a TIMP-1 variant with selectivity for MMP-2 and MMP-9 [100]. The TIMP-2 mutant Ser2Asp/Ser4Ala was effective against MMP-1 (K_i_ = 34 nM), while poorly inhibiting MMP-3, MMP-7, and MT1-MMP [102]. The TIMP-2 mutant Ser4Asp/Ile35Leu/Asn38Ser/Ser68Asp/Val71Ser/His97Ser/Thr99Phe had greatly enhanced specificity for MT1-MMP compared with MMP-9, while the TIMP-2 mutant Ser4Pro/Ile35Pro/Asn38Trp/Ser68Asn/His97Lys/Thr99Lys offered the opposite specificity [103]. Computational and mutational analysis of TIMP-2 interactions with multiple MMPs indicated that TIMP-2 was not optimized to bind to any specific MMP, and thus many affinity-enhancing mutations were possible [104]. Five mutations in TIMP-2, designed to optimize interactions with MT1-MMP (Ile35Met/Asn38Asp/Ser68Asn/Val71Gly/His97Arg), resulted in an inhibitor with K_i_ = 0.9 pM and selectivity over MMP-2 and MMP-10 [105]. While much work has focused on mutations in the N-terminal domain of TIMPs, the C-terminal domain also impacts MMP selectivity. Replacement of the TIMP-1 C-terminal domain with the TIMP-2 C-terminal produced a chimera (T1:T2) that was a much more effective inhibitor of MT1-MMP and MMP-19 than the wild-type TIMP-1 [106]. Subsequent studies led to the identification of residues in both the *N*- and *C*-termini of TIMP-1 that impacted binding to MMP-3 [107]. The importance of cooperativity between TIMP-1 *N*- and *C*-terminal domains was clearly demonstrated, and set the stage for additional considerations in the design of mutant TIMPs as selective metalloproteinase inhibitors. TIMP-2 mutagenesis has been used to engineer a bi-functional protein that binds to the α_v_β_3_ integrin with K_D_ = 130 nM and inhibited MT1-MMP with K_i_ = 77 pM [108]. Concerns exist about the short half-life of TIMPs in vivo, but conjugation of 20 kDa mPEG chains to TIMP-1 Lys residues dramatically extended the half-life in mouse plasma (from 1.1 to 28 h), yet did not significantly impact inhibitory activities either in vitro or in vivo [109]. An additional concern raised about TIMP-based inhibitors is in their ability to penetrate cartilage [110], as needed for osteoarthritis applications.

There are multiple examples of MMP-13 small molecule inhibitors that do not interact with the active site Zn^2+^, but rather bind via so called secondary binding/exosites or allosteric sites [78,111]. Many of these inhibitors take advantage of the MMP-13 P_1_’ subsite. However, distinct drawbacks to these inhibitors have been reported. For inhibitors presented as organic anions, binding to human organic anion transporter 3 resulted in nephrotoxicity [112]. Inhibitors possessing carboxylic acids may generate reactive metabolites through protein conjugation of the resulting acyl glucuronide [112,113]. Pyrimidine-2-carboxamide-4-one-based inhibitors have exhibited poor bioavailability, low volume of distribution, poor metabolic stability, and/or P450 3A4 inhibition [114]. Obtaining appropriate kinetic solubilities for MMP-13 inhibitors has proven challenging [115,116].

**Figure 4:**
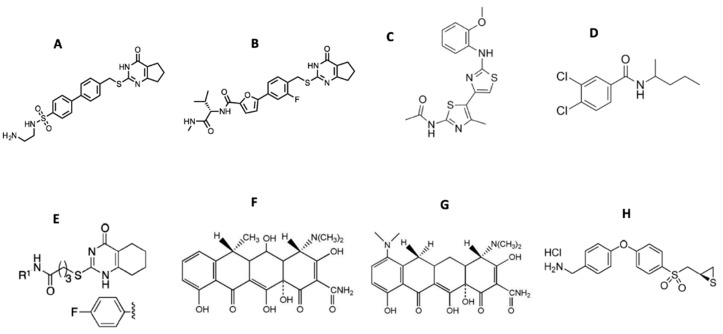
Structures of small molecule MMP inhibitors (**A**) **10d**, (**B**) **(*S*)-17b**, (**C**) JNJ0966 [*N*-(2-((2-methoxyphenyl)amino)-4’-methyl-[4,5’-bithiazol]-2’-yl)acetamide], (**D**) NSC405020 [3,4-dichloro-*N*-(1-methylbutyl)benzamide], (**E**) *N*-(4-fluorophenyl)-4-(4-oxo-3,4,5,6,7,8-hexahydroquinazolin-2-ylthio)butanamide, (**F**) doxycycline, (**G**) minocycline, and (**H**) (*R*)-ND-336.

Starting from the 2-(arylmethylthio)-cyclopentapyrimidin-4-one scaffold identified in a high throughput screen [115,117,118], we synthesized more than 130 compounds with **10d** and **(*S*)-17b** (as numbered when initially published) emerging as the two most promising inhibitors (Figure 4) [119,120]. Both compounds inhibited MMP-13 with low nanomolar IC_50_ values and exhibited an excellent selectivity profile within the MMP family (>170-fold and >1000-fold selectivity, respectively, compared to MMP-1, MMP-2, MMP-9, and MT1-MMP) [119]. Compound **(*S*)-17b** had a long plasma half-life (T_1/2_ = 2.93 h) and a low clearance rate (Cl = 0.18 mL/min/kg) after IV administration in rats [120]. Due to design considerations based on activity profiles and prior data, **10d** and **(*S*)-17b** avoid many of the aforementioned pitfalls of MMP-13 inhibitors, particularly poor solubility and metabolic stability, as well as the potential for nephrotoxicity and generation of reactive metabolites.

Compound JNJ0966 [*N*-(2-((2-methoxyphenyl)amino)-4’-methyl-[4,5’-bithiazol]-2’-yl)acetamide] (Figure 4) inhibited the activation of proMMP-9 and the migration of HT1080 cells [121]. Intratumoral injections of compound NSC405020 [3,4-dichloro-*N*-(1-methylbutyl)benzamide] (Figure 4), an MT1-MMP HPX domain binder, reduced MCF7-β3/MT tumor xenograft size significantly [122]. Screening of oligomer libraries comprised of peptoid and peptide tertiary acid units led to the identification of chemically diverse MT1-MMP inhibitors [123]. One drawback is that these aforementioned compounds typically possess only modest affinities.

As discussed earlier, hydroxylamine was utilized as a template to create MMP inhibitors possessing hydroxamic acid moieties. Hydroxamic acids bind Zn^2+^ in a bidentate fashion [44]. Carboxylic acids bind Zn^2+^ in a monodentate fashion, and thus are more weakly bound to the MMP active site Zn^2+^ than hydroxamic acids [44]. The weaker binding to Zn^2+^ could reduce nonspecific inhibition. Phosphinic acids bind Zn^2+^ in a monodentate fashion, as one phosphinic oxygen binds the MMP active site Zn^2+^ while the second phosphinic oxygen hydrogen bonds to the active site Glu [44]. In addition to a possible advantage due to weaker Zn^2+^ binding, phosphinic acids may have improved metabolic stability compared with hydroxamic acids [48].

A variety of phosphorus-based peptides have been developed as MMP inhibitors, including phosphonates/phosphonic acids [124,125,126,127,128,129,130,131], phosphoramidates [132,133,134], phosphonamidates [133,135,136], and phosphinates/phosphinic peptides [136,137,138,139,140,141,142,143,144,145,146,147,148]. Phosphinates/phosphinic peptides contain a hydrolytically stable, tetrahedral phosphinic pseudo-dipeptide moiety which mimics the tetrahedral intermediate formed during Zn^2+^ metalloproteinase-catalyzed amide bond hydrolysis. Phosphinic peptides offer the possibility of interacting with both the primed and unprimed sides of the MMP active site cleft [43]. We have reported that phosphinic triple-helical peptides (THPs) behave as effective transition state analog inhibitors of collagenolytic MMPs [67,149,150,151,152]. These triple-helical peptide inhibitors (THPIs) have proven effective in mouse models of multiple sclerosis, sepsis, and myocardial infarction [67,153]. THPs have been found to be reasonably stable to general proteolysis, as observed in vitro in mouse, rat, and human serum and/or plasma and in vivo in mice and rats [154,155,156,157,158,159]. THPs were found to exhibit high stability (72%) when administered orally to rats [160].

Function blocking metallobodies (SDS3 and SDS4) were generated by immunization directed at the catalytic Zn^2+^ and enzyme surface epitopes in activated MMP-9 [161]. SDS3 was shown, in both prophylactic and therapeutic applications, to protect mice from dextran sodium sulfate-induced colitis [161].

Numerous MMP inhibitory antibodies have been developed [23,26,95,162]. Mouse mAb REGA-3G12, a selective inhibitor of MMP-9 [163], prevented interleukin-8-induced mobilization of hematopoietic progenitor cells in rhesus monkeys [164]. Two monoclonal anti-MMP-9 antibodies, AB0041 and AB0046, were shown to inhibit tumor growth and metastasis in a model of colorectal carcinoma [72]. A humanized version of AB0041, GS-5745/andecaliximab, was generated for use in clinical trials [72]. As mentioned earlier, GS-5745 inhibited proMMP-9 activation and non-competitively inhibited MMP-9 activity [76].

DX-2400, a selective, fully human MT1-MMP antibody inhibited metastasis in a breast cancer xenograft mouse model [165]. DX-2400 also enhanced tumor response to radiation therapy [166]. Two humanized scFv antibodies, CHA and CHL, generated against the MT1-MMP HPX domain inhibited HT1080 invasion of type I collagen [167]. Human scFv-Fc antibody E3 bound to the MT1-MMP CAT domain and inhibited type I collagen binding [168]. A second generation E3 clone (E2_C6) inhibited tumor growth and metastasis [168]. Human antibody Fab libraries were synthesized, where the Peptide G sequence (Phe-Ser-Ile-Ala-His-Glu) [169] was incorporated into complementarity-determining region (CDR)-H3 [170]. The resulting Fab 1F8 inhibited MT1-MMP CAT domain activity [170]. Screening of a phage displayed synthetic humanized Fab library led to the identification of Fab 3369, which inhibited the activity of the MT1-MMP CAT domain [171]. IgG 3369 treatment of MDA-MB-231 mammary orthotopic xenograft mice reduced lung metastases, collagen processing, and tumor density of CD31^+^ blood vessels [171].

mAb 9E8 inhibited MT1-MMP activation of proMMP-2, but not other MT1-MMP catalytic activities [172]. Antibody LOOP_Ab_ also inhibited MT1-MMP activation of proMMP-2 but not MT1-MMP collagenolysis [173]. These antibodies represent approaches in which a *specific activity* of an MMP is inhibited, which would prove especially beneficial in cases where maintaining certain activities of the MMP are desirable to support normal physiological functions.

The LEM-2/15 antibody was generated using a cyclic peptide mimicking the MT1-MMP CAT domain V-B loop (residues 218-233) [174]. A minimized Fab fragment of LEM-2/15 [175] significantly increased the ability of virally infected mice to fight off secondary *Streptococcus pneumoniae* bacterial infection [176].

It has been noted that antibody antigen binding sites are not complementary to the concave shape of catalytic clefts, as antigen binding sites are planar or concave [95]. To overcome this, the convex-shaped paratope of camelid antibodies was incorporated into the human antibody scaffold [177]. The resulting Fab 3A2 was a competitive MT1-MMP inhibitor which reduced metastasis in a melanoma mouse model [178].

There are several inhibitors that act by disrupting interactions of MMPs with cell surface binding partners. The MMP-9 HPX domain binds to B chronic lymphocytic leukemia (B-CLL) cells via the α4β1 integrin [179]. A variant of the MMP-9 HPX domain containing blades III and IV (B3B4) interacted with the α4β1 integrin, and a peptide derived from this region (peptide P3, residues 654-674, PFPGVPLDTHDVFQYREKAYFC) inhibited cell adhesion. A truncated version of peptide P3 (P3a, FPGVPLDTHDVFQYREK) inhibited B-CLL and MEC-1 cell adhesion to proMMP-9 and inhibited B-CLL cell transendothelial migration and intracellular survival signals. The compound *N*-(4-fluorophenyl)-4-(4-oxo-3,4,5,6,7,8-hexahydroquinazolin-2-ylthio)butanamide (Figure 4) prevented association of proMMP-9 with the α4β1 integrin and CD44, resulting in the dissociation of epidermal growth factor receptor (EGFR) from the β1 integrin subunit and CD44 [180].

Interaction of CD44 with MT1-MMP was found to require the outermost strand of HPX domain blade I [181]. A peptide derived from this blade, IS4 (acetyl-VMDGYPMP-NH_2_), inhibited MT1-MMP mediated cell migration and metastasis in vivo [181].

### 3.2. Timing of MMP Inhibition

Often overlooked is that the timing of MMP inhibitor application may be critical to achieve the desired therapeutic effect. Application of marimastat in combination with gemcitabine significantly improved survival in pancreatic cancer patients with disease confined to the pancreas [182]. Presurgical treatment with the oral MMP inhibitor SD-7300 (*N*-hydroxy-1-(2-methoxyethyl)-4-[4-[4-(trifluoromethoxy)phenoxy]piperidin-1-yl]sulfonylpiperidine-4-carboxamide, with high inhibitory activity towards MMP-2, MMP-9, and MMP-13) improved survival from 67 to 92% in a mouse breast cancer model and significantly decreased the risk of recurrence [183]. The “window of opportunity” for MMP inhibitor application in cancer is often in premetastatic disease [30,184,185,186]. In the future, MMP inhibitors may need to be considered in metastasis prevention trials.

MMP-9 is a key contributor to the “angiogenic switch” during carcinogenesis of pancreatic islets [187]. However, MMP-9 deficiency in pancreatic ductal adenocarcinoma (PDAC) mouse models resulted in more invasive tumors and an increase in desmoplastic stroma [188]. The absence of MMP-9 led to increased interleukin 6 levels in the bone marrow, which activated tumor cell STAT3 signaling and promoted PDAC invasion and metastasis [188]. Thus, MMP-9 represents an anti-target in the later stage of pancreatic cancer.

### 3.3. Side Effects of Selective MMP Inhibitors

The monoclonal anti-MMP-9 antibody GS-5745/andecaliximab has been evaluated under several clinical trials. The outcome of a randomized placebo controlled phase 1b single and multiple ascending dose-ranging clinical trial on 72 patients diagnosed with moderately to severely active UC showed that GS-5745/andecaliximab was safe and well tolerated [73]. A phase 2/3 UC study with 165 patients treated over 8 weeks further indicated that GS-5745/andecaliximab was well tolerated [74]. A phase 1b trial investigating the safety, pharmacokinetics, and disease-related outcomes for 15 rheumatoid arthritis patients demonstrated that GS-5745/andecaliximab was safe, with adverse events that were only grade 1 or 2 in severity and no indication of MSS [189]. A phase 1 GS-5745/andecaliximab dose-finding trial of 13 patients with advanced solid tumors found no dose-limiting toxicity at any concentration (200, 600, or 1800 mg IV every 2 weeks), with adverse events that were only grade 1 or 2 in severity [190]. These clinical trials provide further evidence that selective MMP inhibitors can be developed without inducing MSS.

## 4. The Outlook for Current MMP Inhibitors

The lack of success in early MMP clinical trials has created a strong negative bias toward MMP inhibition as a clinical strategy. In actuality, MMP clinical trials were undertaken prematurely [191,192], due to insufficient knowledge of MMP biology and the choice of hydroxamic acid as the active component of the inhibitors. Unique modes of action have been used to develop the next generation of MMP inhibitors. Clinical trials utilizing antibodies have provided evidence that selective MMP inhibitors do not induce MSS. The therapeutic potential of anti-MMP antibodies has yet to be realized, with one concern being limited extravascular distribution [189,193]. Antibodies are also subject to proteolysis, may be removed from circulation rapidly, and are costly. Nonetheless, antibodies have provided truly selective, high affinity MMP inhibitors. In addition, selective, high affinity inhibitors can be developed for MMPs based on triple-helical structure. Triple-helical peptide inhibitors (THPIs) have excellent pharmacokinetic properties compared with other peptide-based therapeutics.

### 4.1. MMP Inhibition and the Immune System

It has been proposed that the major role of MMPs is in modulation of inflammatory and immune processes [17,79]. MMP processing can either activate or inhibit a variety of chemokines and cytokines [79,194]. For example, MMP-9 cleaves interleukin-8 and increases the activity of the chemokine 10-fold for neutrophil activation and chemotaxis [195]. In turn, neutrophils release a TIMP-free form of MMP-9 which promotes angiogenesis [196]. Overall, MMPs can cleave virtually all human chemokines [197]. A better understanding of MMPs in the immune system and in inflammatory diseases may be necessary before proceeding with cancer-based clinical trials [191].

Insight into the roles of MMPs in the immune system has revealed potential applications of MMP inhibitors to improve immunotherapy. Monoclonal MMP-9 antibody AB0046, which inhibited tumor growth and metastasis in a surgical orthotopic xenograft model of colorectal carcinoma [72], improved immune responses to tumors, as the inhibition of MMP-9 reversed MMP-9 inactivation of T-cell chemoattractant CXCR3 ligands (CXCL9, CXCL10, and CXCL11) [198]. MT1-MMP shed tumor cell MHC class I chain-related molecule A (MICA) [199]. Engagement of MICA to NKG2D stimulated natural killer (NK) and T-cell antitumor activity [199]. Protection of MICA stimulated antitumor immunity and reduced metastasis in a humanized melanoma mouse model [200].

Examination of tumor cryosections in an MT1-MMP antibody treated MDA-MB-231 triple-negative breast cancer xenograft mouse model revealed an increased density of iNOS+ cells (a marker of anti-tumor M1 tumor-associated macrophages) and Granzyme B+ cells [171]. Application of an MT1-MMP antibody to a 4T1 triple-negative breast cancer mouse model shifted macrophages towards the antitumor M1-like phenotype and reduced activated TGFβ (an immunosuppressive cytokine) [166].

### 4.2. MMP Inhibitors in the Clinic and in Clinical Trials

There is an established history of clinically successful MMP inhibitors. Periostat, which is doxycycline (Figure 4) formulated at a sub-antimicrobial dose, is a U.S. Food and Drug Administration (FDA)-approved MMP inhibitor for periodontal disease treatment [201,202,203,204]. Doxycycline is a broad-spectrum MMP inhibitor with IC_50_ values in the range of 2–50 μM [18]. Tetracyclines have shown promise in other MMP indications. Treatment of 16 relapsing–remitting multiple sclerosis patients with a doxycycline/interferon combination for 4 months (ClinicalTrials.gov Identifier NCT00246324) reduced brain lesions and MMP-9 serum levels and improved Expanded Disability Status Scale values [205]. The treatment was considered effective, safe, and well tolerated [205,206]. In other trials for relapsing–remitting multiple sclerosis, minocycline (Figure 4) treatment resulted in no new active lesions after 1 month with detection up to 6 months for 10 patients [207] while conversion from a first demyelinating event (clinically isolated syndrome) to multiple sclerosis was lowered by 18.5% over 6 months but was not lowered over 24 months for 72 patients [208]. Combination treatment of minocycline with glatiramer acetate, compared with glatiramer alone, in 44 patients showed the combination therapy reduced the number of lesions by 63–65% and lowered the risk of relapse after 8–9 months [209]. The treatment was considered safe and well tolerated [209]. Additional clinical trials using minocycline for multiple sclerosis treatment are reportedly upcoming [6]. In similar fashion to doxycycline, minocycline is a broad-spectrum MMP inhibitor, with IC_50_ values in the 100–300 μM range [18].

Minocycline has also been examined for improving neurological outcomes following stroke [210,211]. MMP-9 levels are increased following cerebral ischemia, and are further enhanced by tissue plasminogen activator (tPA) stroke treatment [212,213,214]. Administered within 6 h of stroke symptom onset in 60 patients, minocyline was well tolerated up to 10 mg/kg intravenous administration and had a half-life of 24 h [210]. Minocycline decreased MMP-9 levels at 24 h and to baseline in 72 h in 36 patients treated with tPA [211]. Incyclinide (a.k.a. Metastat, COL-3), a chemically modified tetracycline with no antibiotic activity, showed a significant regression of AIDS-related Kaposi’s sarcoma with a significant reduction of MMP-2 and MMP-9 plasma levels [215].

Clinical trials using anti-MMP antibodies have moved forward. GS5745/andecaliximab in combination with mFOLFOX6 (oxaliplatin, leucovorin, and 5-fluorouracil) was utilized in a phase I clinical trial of 40 patients with HER2-negative gastric and gastroesophageal junction adenocarcinoma [190]. The overall response rate for all patients was 47.5% (7.5% complete response and 40% partial response), with no observed MSS [190]. As described earlier, one concern about GS5745/andecaliximab is the much higher affinity for proMMP-9 compared with active MMP-9 [76].

Topical application of MMP inhibitors may also prove to be efficacious. In human diabetic foot ulcers, levels of active MMP-8 and MMP-9 are increased [216]. MMP-9 has been identified as a limiting factor in healing, while MMP-8 facilitates wound repair [20,64,216]. The compound (*R*)-ND-336 (Figure 4) selectively inhibits MMP-2, MMP-9, and MT1-MMP with K_i_ < 100 nM, while weakly inhibiting MMP-8 [216]. Thus, (*R*)-ND-336 has the desired inhibitory profile for application to diabetic foot ulcers. Additionally, (*R*)-ND-336 is a slow binding inhibitor of MMP-2, MMP-9, and MT1-MMP with a long residence time [216]. (*R*)-ND-336 is more efficacious than becaplermin in animal models of wound healing, and thus represents a promising topical treatment for diabetic foot ulcers [64,216]. Clinical trials for (*R*)-ND-336 utility are anticipated (https://ideacenter.nd.edu/news-events/news/new-hope-for-the-treatment-of-diabetic-foot-ulcers/).

A related, promising therapeutic approach is the use of MMPs to activate prodrugs and imaging agents or facilitate drug delivery [27,217,218,219,220,221]. A concern with these approaches is the “selectivity” of the peptide sequences used for activation [217,218,219,222]. ICT2588 is a vascular disrupting agent designed to be activated by MT1-MMP, containing an Arg-Ser-Cit-Gly~Hof-Tyr-Leu-Tyr sequence (where Cit is citrulline and Hof is homophenylalanine) with azademethylcolchicine at the *C*-terminus and fluorescein (FITC) at the *N*-terminus [223,224]. Azademethylcolchicine functions as the vascular disrupting agent. Following hydrolysis at the Gly~Hof bond by MT1-MMP, exopeptidase activity removes the remaining Hof-Tyr-Leu-Tyr sequence to liberate azademethylcolchicine [223]. ICT2588 produced a 90% decrease in functional tumor vasculature in HT1080 tumor-bearing mice, and co-administration of ICT2588 and doxorubicin was significantly more effective in reducing tumor volume compared with either ICT2588 or doxorubicin alone [223]. Conjugation of a variant of ICT2588 (FITC-βAla-Cys-Arg-Ser-Cit-Gly~Hof-Tyr-Leu-Tyr-azademethylcolchicine) with a cross-linked iron oxide (CLIO) nanocarrier resulted in a theranostic that allowed for magnetic resonance imaging of drug delivery and accumulation in tumors [220,225]. Co-administration of the CLIO-ICT with temozolomide resulted in decreased tumor size and increased survival in glioblastoma xenograft mice [226]. ICT2588 was scheduled to begin Phase 1 clinical trials in 2018 (http://www.incanthera.com/what-we-do/technology-pipeline/).

## 5. Conclusions

There is considerable promise in the recent generation of MMP inhibitors. Valuable lessons were learned from prior clinical trial failures, including the need for selective and metabolically stable inhibitors. Proteomics approaches have better defined the impact of MMP activity on biological systems. Short-term indications may be needed when anti-target activities are of concern. While there is great reliance on mouse models for examining MMP in vivo behaviors, it has been recognized that animal models may not clearly reflect human conditions and mechanism of disease. Consideration of MMP secondary binding sites (exosites) offers the best opportunity for development of selective inhibitors. The FDA approval of an MMP inhibitor and the advancement of numerous MMP inhibitors to clinical trials clearly indicates that it is time to depart from the dogma of viewing MMP inhibition as intractable.
